# Severe Maxillary Protrusion Treated with Surgically Assisted Rapid Maxillary Expansion

**DOI:** 10.3390/jcm13144149

**Published:** 2024-07-16

**Authors:** Sonoko Okuhashi, Masaru Kobayashi, Eiji Tanaka

**Affiliations:** 1 Department of Craniofacial Growth and Development Dentistry, Division of Orthodontics, Kanagawa Dental University, Yokosuka 238-8580, Japan; sonoko@cube.ocn.ne.jp; 2 Private Practice of Orthodontics, Sagamihara 252-0303, Japan; 3 Department of Oral and Maxillofacial Surgery, Kanagawa Dental University, Yokosuka 238-8580, Japan; m.kobayashi@kdu.ac.jp; 4 Department of Orthodontics and Dentofacial Orthopedics, Tokushima University Graduate School of Biomedical Sciences, Tokushima 770-8504, Japan

**Keywords:** surgically assisted rapid maxillary expansion, maxillary protrusion, maxillary group distalization, Anchor-Lock system

## Abstract

In this case, surgically assisted rapid maxillary expansion (SARME) was successfully adopted to treat a skeletal maxillary protrusion with large overjet and severe crowding. The female patient, aged 25 years and 11 months, was diagnosed with skeletal maxillary protrusion with severe crowding and excessive overjet associated with labially inclined maxillary central incisors. After achieving sufficient space for surgical incision between bilateral maxillary central incisors, the SARME was performed. A total of 8.0 mm lateral expansion of the maxilla was completed. At 48 days after surgery, the Hyrax appliance was replaced with an Anchor-Lock system used as an external surgical stent and skeletal anchorage for maxillary group distalization, and the distal movement of the maxillary molars was initiated without waiting for bone healing of the separated midpalatal suture by SARME. Twenty-five months’ treatment, including surgical preparation, achieved an acceptable and stable occlusion with adequate interincisal relationship. The occlusion was much more stable with a little relapse through more than 4 years’ retention period. In conclusion, SARME followed by the Anchor-Lock system might lead to favorable occlusal outcome in the long term without any relapses.

## 1. Introduction

Rapid maxillary expansion (RME) is an effective method to alleviate crowding, correct posterior crossbite, and transversely expand the maxilla [1], and it is commonly accepted as the standard procedure especially for patients who are growing [2,3,4]. However, RME is contraindicated in post-pubertal patients because craniofacial sutures enhance their stiffness and interdigitation with aging [5], leading to a high failure rate in mature patients [6]. For this reason, the most commonly used procedures to expand narrower maxillae in post-pubertal patients are surgically assisted rapid maxillary expansion (SARME) or miniscrew-assisted palatal expansion (MARPE) [7,8,9,10].

Since SARME was first described as a midpalatal splitting in 1938, a variety of SARME modalities have been reported and various treatment cases using SARME have been published [11,12]. SARME was first developed for the purpose of accelerating orthodontic tooth movement, and thereby reducing treatment duration. SARME is an important method for the correction of moderate-to-severe maxillary transverse deficiencies in non-growing patients with a greater degree of stability and without disadvantages for periodontal health [13]. On the contrary, several disadvantages have been reported: median diastema, median papilla infringement, root and pulp damage at the osteotomy area, numbness of lip and palate, and risk of nasal septum deviation [14]. To date, no standard protocol for SARME has been developed [12,15,16]. Magnusson et al. [17] suggested that the reduction in maxillary transverse width after SARME probably occurs during the first 3 years post-surgery and that it is stable within 6 years post-surgery. This implies that longer retention of the expanded maxilla leads to a more stable outcome, while the expansion device should remain in place until bone healing is achieved. In the meantime, it is difficult to continue orthodontic treatment.

For this reason, an external palatal plate, the Anchor-Lock System (Compact Lock 2.0, Johnson & Johnson Corp., New Brunswick, NJ, USA), was developed to maintain the expanded maxillary width and simultaneously distalize the maxillary molars [18,19]. With the use of this system, we can initiate distal movements of the maxillary molars without waiting for sufficient latency period after the skeletal expansion of the maxilla by SARME. This leads to a reduction in treatment duration.

The aim of this article was to show a case of severe maxillary protrusion with a narrow maxilla and excessive overjet that was successfully treated with SARME followed by the Anchor-Lock system.

## 2. Detailed Case Description

**Figure 1:**
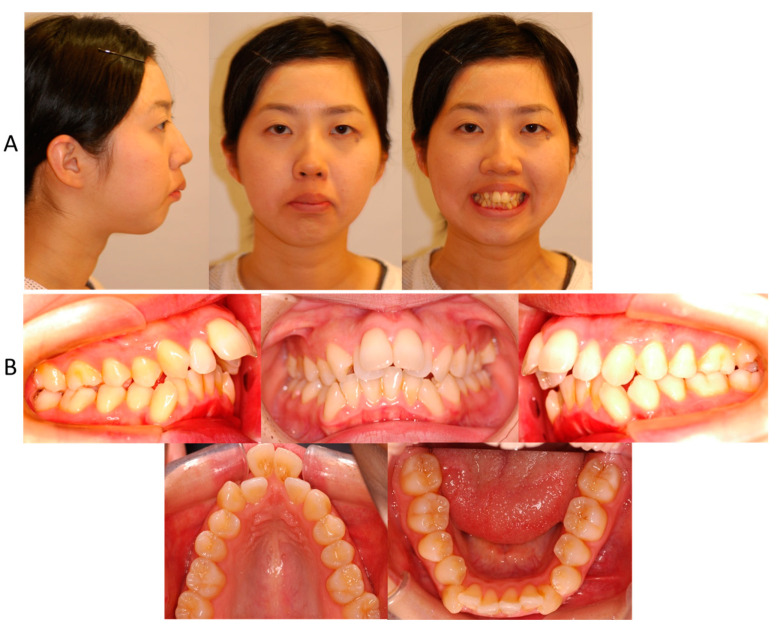
Facial (**A**) and intraoral (**B**) photographs before treatment at the age of 25 years and 11 months.

Informed consent was obtained from the patient for this case report. The patient, a female, 25 years and 11 months olde, had chief complaints of maxillary protrusion with narrow maxillary arch resulting in difficulty closing the lips. The patient exhibited no significant medical history. Furthermore, there was no history of trauma to the head, neck, or jaw. The facial profile was convex due to a retropositioned chin with lip protrusion, and the frontal face was asymmetric, with a slight rightward shift of the chin (Figure 1). The first molar relationship was Angle Class II on the right side, while the left side showed Angle Class I. The overjet and overbite were 16.0 mm and 3.0 mm, respectively (Figure 1). The maxillary dental arch showed as V-shaped, while the mandibular dental arch was symmetrical square. From the model analysis, both maxillary and mandibular basal arch widths were less than −2 S.D. smaller than the Japanese standard, and both maxillary and mandibular dental arch widths were also significantly smaller [20]. The maxillary intermolar width was 29.0 mm, which is also smaller than the Japanese standard. The arch length discrepancies were −15.3 mm on the maxillary dentition and −7.2 mm on the mandibular dentition. While the upper dental midline was matched up to the facial midline, the mandibular dental midline shifted 4.5 mm to the right of the maxillary dental midline.

**Figure 2:**
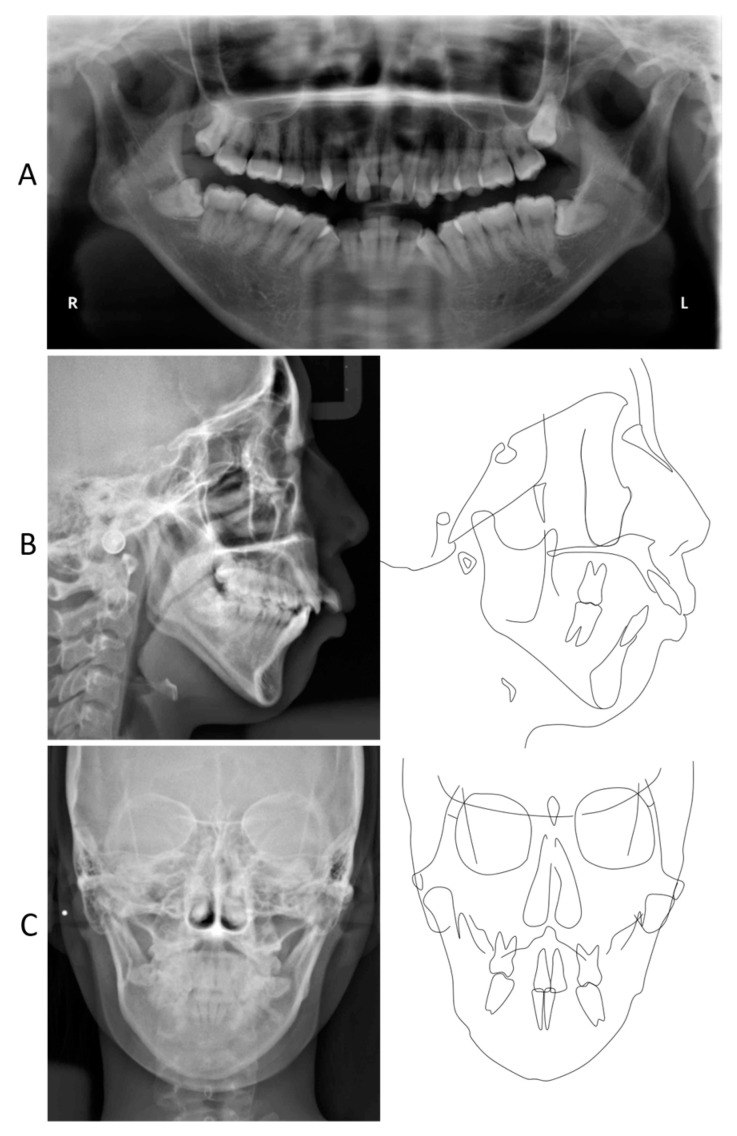
Pretreatment radiographical records. (**A**) Panoramic radiograph; (**B**) Lateral cephalogram and its tracing; (**C**) Frontal cephalogram and its tracing.

A panoramic radiograph showed horizontal impaction of the mandibular third molars, while the maxillary right third molar had erupted (Figure 2). Both mandibular condyles showed flattening with short condylar heads, although there were no symptoms of temporomandibular joint disorders. The cephalometric analysis revealed that the skeletal jaw-base relationship of the patient was Class II (ANB, 8.0°) compared to the Japanese norm (Figure 2; Table 1) [21]. The mandibular plane and gonial angles were greater than those of the Japanese control (FMA, 47.0°; Gonial A, 138.0°). The maxillary central incisors were tilted labially (U1-SN, 118.0°), whereas the mandibular central incisal inclination was within the normal range (L1-Mp, 87.0°). The upper and lower lip positions were +7.5 mm and +8.5 mm in relation to the E-line, respectively. A frontal cephalogram showed a mandibular asymmetry with 1.0° of an occlusal cant and 1.5 mm of a rightward shift of the menton (Figure 2).

### 2.1. Treatment Objectives

The patient was diagnosed with a skeletal maxillary protrusion accompanied by a narrow maxillary arch and large overjet caused by excessive labial inclination of maxillary central incisors. The treatment objectives were (1) to correct the labial inclination of the maxillary central incisors involved in the narrow maxilla, (2) to resolve the crowding with the mandibular midline deviation, and (3) to obtain a functional Class I occlusion with proper canine and molar relationships. Treatment was then planned as follows:

1. Expansion of the maxillary arch by 8.0 mm bilaterally with SARME.
2. Miniscrew-assisted distalization of 5.0 mm on the right and 6.0 mm on the left maxillary dentitions.
3. Miniscrew-assisted distalization of 1.0 mm on the right and 4.0 mm on the left mandibular dentitions.

### 2.2. Treatment Alternatives

To expand the maxillary arch is absolutely essential to correct a narrow maxillary arch with a large arch length discrepancy. The first alternative was RME to correct the inadequate width of the maxillary arch and obtain space for teeth alignment. However, maxillary expansion with RME often induces undesirable side effects, especially in non-growing patients, such as gingival recession, root resorption, buccal cortex fenestration, and insufficient maxillary expansion [5,6]. The second alternative is to extract the first premolars and use SARME or MARPE to complete skeletal expansion in matured maxilla. MARPE enables us to avoid surgery, which can cause complications such as infection, post-surgery pain and discomfort, or late relapse [22]. However, as well as RME, MARPE is connected to the screws placed in the midpalatal areas, revealing the expansion force that can be seen on both sides of the suture. This may result in the unilateral overcorrection, leading to unilateral incisors’ bite. Therefore, SARME was designed to expand the maxillary arch bilaterally and correct a significant arch length discrepancy. Because the patient desired non-extraction of premolars, both maxillary and mandibular group distalization was attempted using skeletal anchorage devices. Furthermore, since the patient wanted to reduce the treatment duration as much as possible, we decided to adopt the Anchor-Lock System (Compact Lock, Johnson & Johnson Corp.) for rigid bone fixation after SARME, resulting in no or shorter latency period after SARME.

### 2.3. Treatment Progress

**Figure 3:**
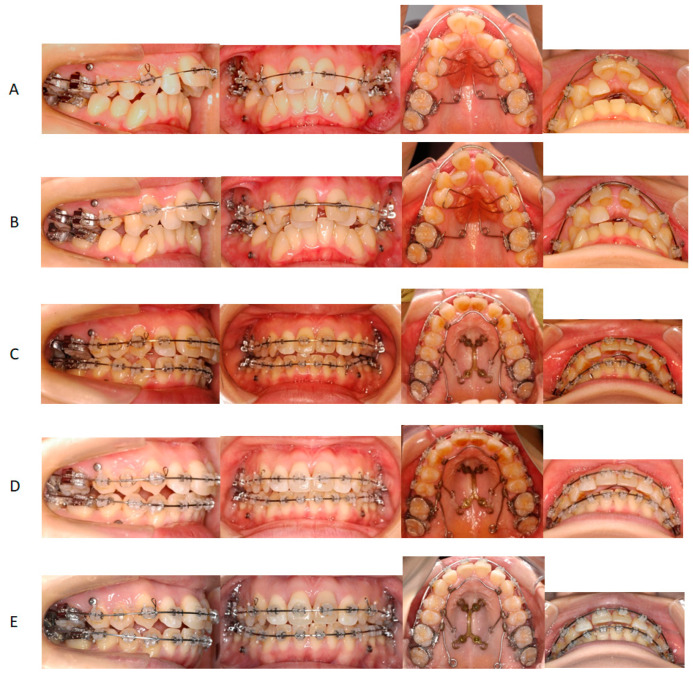
Intraoral photographs during treatment. (**A**) One month; (**B**) Two months; (**C**) Thirteen months (9 months after SARME); (**D**) Sixteen months (12 months after SARME); (**E**) Twenty months (16 months after SARME).

After extraction of the bilateral maxillary and mandibular third molars, 0.022 in slot preadjusted edgewise appliances (MBT-prescription Unitek Clarity Ultra, 3M, Monrovia, CA, USA) were placed on the maxillary dentition except for the bilateral lateral incisors and second premolars, in addition to a pendulum appliance to distalize the bilateral maxillary first molars (Figure 3A). Furthermore, miniscrews were also placed bilaterally between the maxillary second premolars and first molars, and canine retraction was initiated bilaterally. After two months of distalization, a 2 mm space was gained for a safe incision between the maxillary central incisors (Figure 3B).

**Figure 4:**
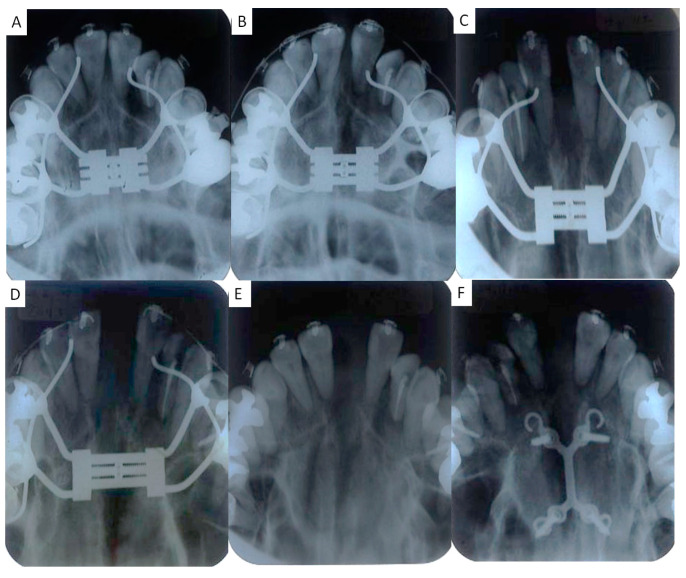
Occlusal radiographs. (**A**) Before SARME; (**B**) Day 6 after SARME; (**C**) Day 11 after SARME; (**D**) Day 25 after SARME (Completion of maxillary expansion); (**E**) Day 48 after SARME (immediately after removal of Hyrax appliance); (**F**) Day 48 after SARME.

**Figure 5:**
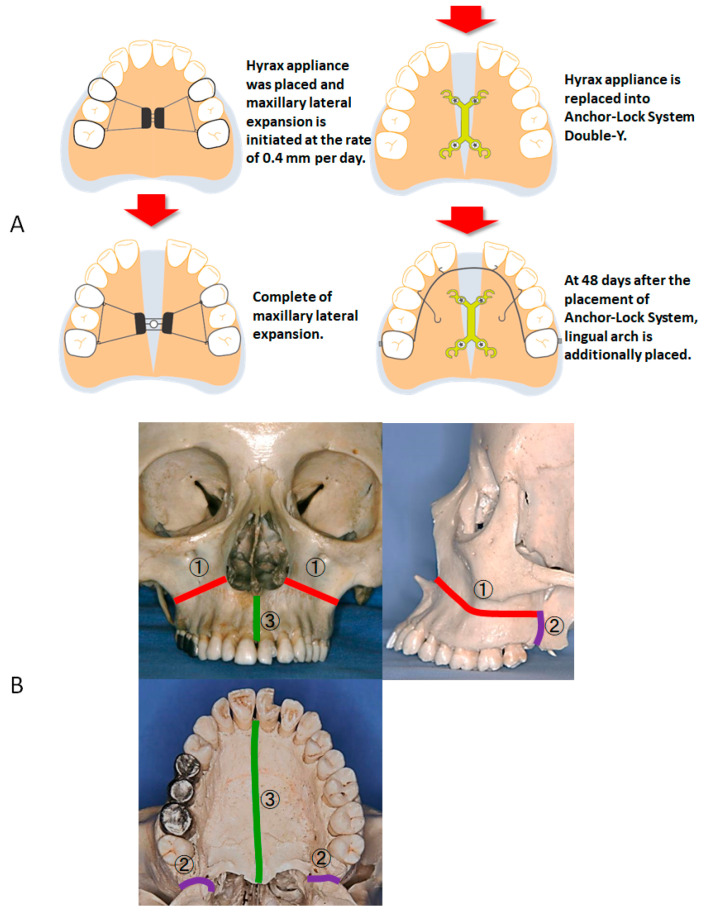
Schematic illustration of SARME treatment procedure with the Anchor-Lock system (**A**) and multisegmental LeFort I osteotomy (**B**). ① Basic osteotomy from the periform aperture to the pterygoid plate; ② Separation of the pterygoid junction; ③ Separation of the midpalatal suture.

**Figure 6:**
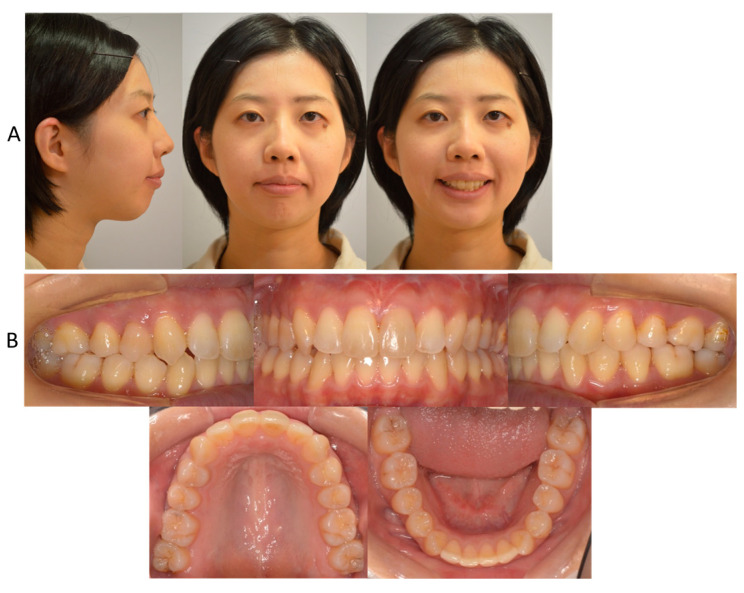
Posttreatment facial (**A**) and intraoral (**B**) photographs at the age of 28 years and 4 months.

Then, the Hyrax appliance was replaced with a pendulum appliance onto the maxillary arch, and SARME was carried out (Figure 4A and Figure 5A). Under general anesthesia, multisegmental LeFort I osteotomy was performed from the piriform aperture posteriorly to the pterygoid plate with separation of the pterygoid junction (Figure 5B). This osteotomy was conducted 5 mm above the roots of the premolars (Figure 5B). The second osteotomy was performed vertically between the central incisors, followed by an osteotomy in the maxillary midpalatal suture (Figure 6B). Completion of the separation was confirmed from the occlusal radiographs taken before, after 6 days, 11 days, 25 days, and 48 days of SARME (Figure 4B–E and Figure 5A). After 5 days of the latency period, the expansion of the maxilla was started at the rate of 0.4 mm per day. A total of 8.0 mm lateral expansion was completed on day 25 after SARME (Figure 4D and Figure 5A). At 48 days after surgery, the Hyrax appliance was removed and replaced with a double-Y-shaped external plate (Lock plate, Double-Y Type) to stabilize the maxillary expansion and initiate the maxillary group distalization (Figure 4F and Figure 5A). The double-Y-shaped external plate was fixed with a total of 4 titanium screws: two screws of 12.0 mm length and 2.0 mm diameter in the anterior plate and two screws of 10.0 mm length and 2.0 mm diameter in the posterior plate. A 0.045 in stainless steel palatal arch with hooks was connected to the first molars, and elastometric chains were placed between the Y-plate and the hooks to accelerate the maxillary group distalization (Figure 3C). The miniscrews placed on the buccal interradicular area between the maxillary second premolars and first molars were used to intrude the maxillary molars. At 16 months of treatment after surgery, the maxillary right and left molars were distalized to 5.0 mm and 6.0 mm, respectively, resulting in improving severe anterior crowding and excessive overjet without premolars’ extraction (Figure 3E).

The miniscrews were bilaterally inserted between the mandibular first and second premolars, and 2 N force was applied to the bilateral mandibular canine by elastometric chains (Figure 3D). During 16 months of treatment with a multibracket appliance, 1.0 mm and 4.0 mm distalization were achieved on the right and left sides, respectively.

After 25 months of multibracket treatment including 4 months preparation for SARME, an acceptable occlusion was obtained. After the removal of the appliances, a wraparound-type retainer was placed on both maxillary and mandibular dentitions.

### 2.4. Treatment Results

After active orthodontic treatment, the patient obtained a balanced facial profile with properly positioned upper and lower lips (Figure 6). The maxillary and mandibular dental midlines were coincident with the facial midline (Figure 6). Her lips showed less lip closure tension. The upper and lower lip positions were +1.0 mm and +2.0 mm in reference to E-line, respectively. Stable intercuspation of the teeth was obtained with Class I canine and molar relationships (Figure 6). The overjet was improved to 2.5 mm, and the overbite was well maintained at 2.5 mm. The maxillary intermolar width increased from 29.0 mm to 36.0 mm.

**Figure 7:**
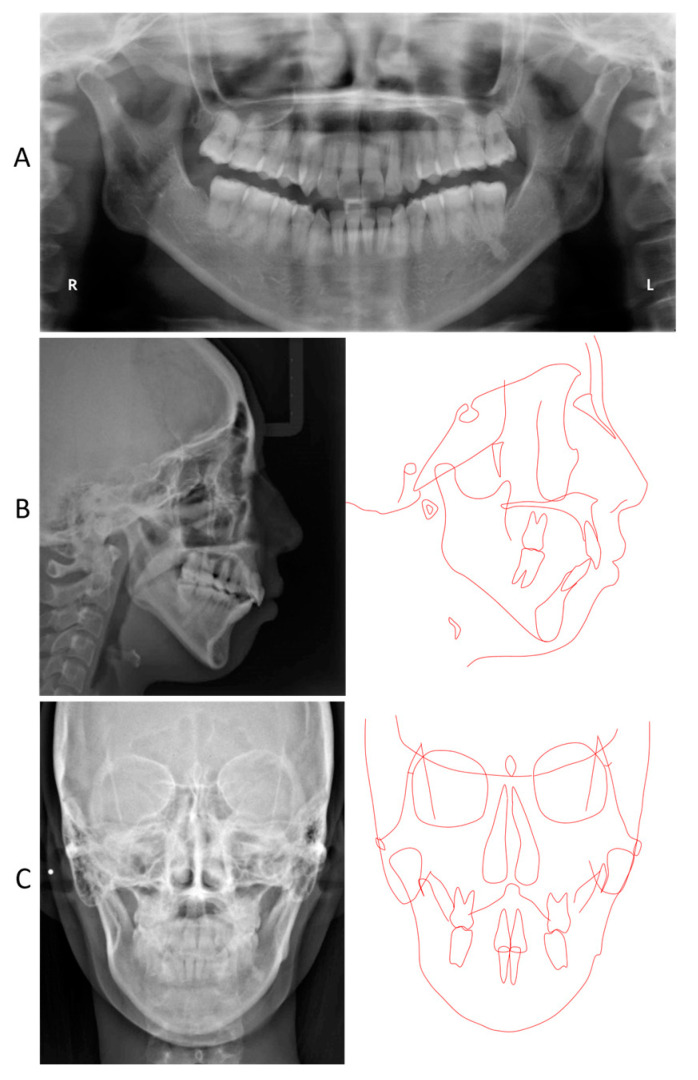
Posttreatment radiographical records. (**A**) Panoramic radiograph; (**B**) Lateral cephalogram and its tracing; (**C**) Frontal cephalogram and its tracing.

**Figure 8:**
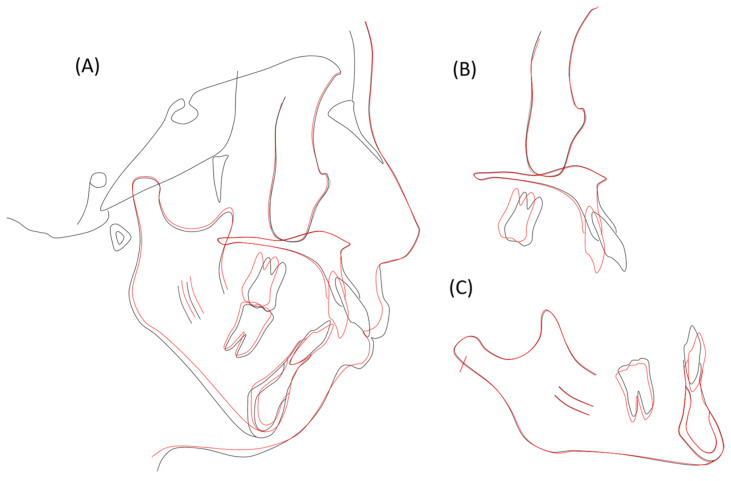
Pretreatment (black; age 25 years, 11 months) and posttreatment (red; age 28 years, 4 months) cephalometric tracings superimposed on the internal contour of the anterior wall of the sella turcica (**A**), the anterior contour of the zygomatic process (**B**), and the internal contour of the cortical plate at the inferior border of the symphysis (**C**).

The panoramic radiograph revealed proper root parallelism (Figure 7). No or minimal root resorption was detected in the maxillary anterior teeth. Posttreatment cephalometric analysis revealed a skeletal Class II relationship (ANB, 7.0°), although the ANB angle decreased by 1.0°, and the SNB angle increased by 1.0°. The mandibular plane angle decreased by 1.5°, caused by the mandibular counterclockwise rotation; however, she was still classified as a high mandibular plane angle case (FMA, 45.5°), the same as pretreatment (Figure 7 and Figure 8; Table 1). The maxillary first molars were distalized 5.0 mm on the right and 6.0 mm on the left, and intruded 2.0 mm on both sides. The mandibular first molars were also moved distally by 0.5 mm on the right and 4.0 mm on the left. The maxillary and mandibular central incisors were tilted lingually by 30.0° and labially by 3.0°, respectively, leading to a proper interincisal angle (IIA, 130.0°). A frontal cephalogram indicated mandibular symmetry with a coincidence of the maxillary and mandibular dental midlines and amelioration of a right-side shift of the chin (Figure 7).

**Figure 9:**
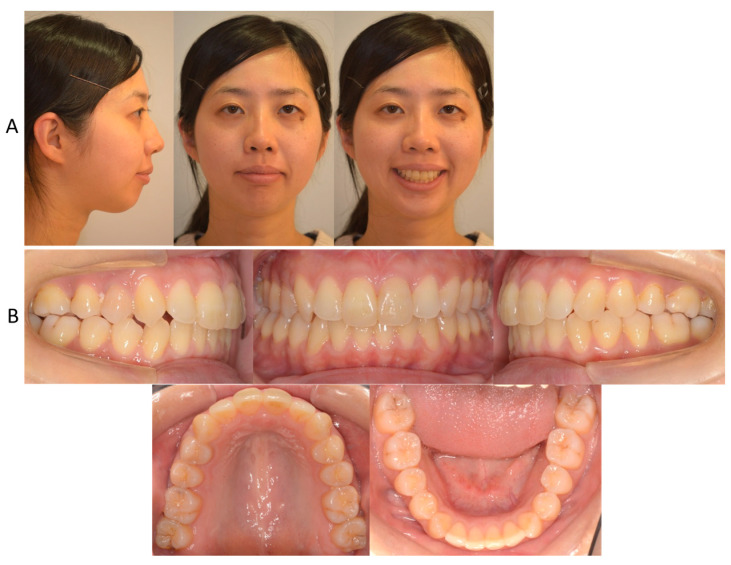
Facial (**A**) and intraoral (**B**) photographs after active treatment at the age of 32 years and 6 months.

**Figure 10:**
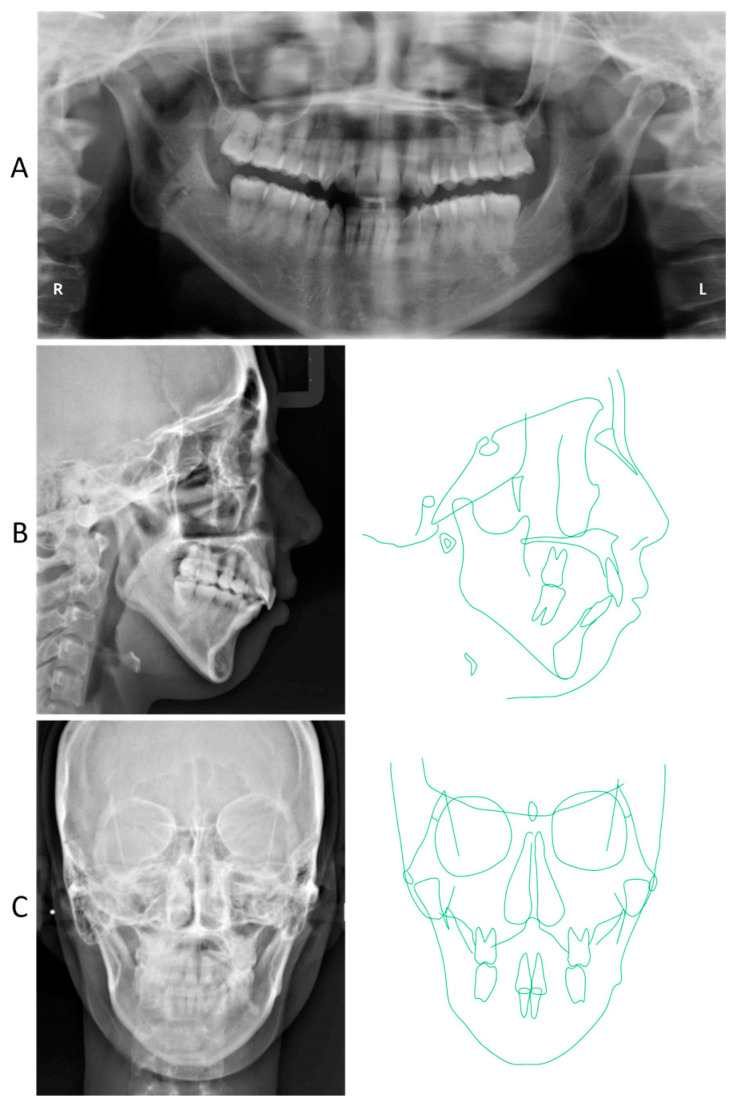
Postretention radiographical records. (**A**) Panoramic radiograph; (**B**) Lateral cephalogram and its tracing; (**C**) Frontal cephalogram and its tracing.

**Figure 11:**
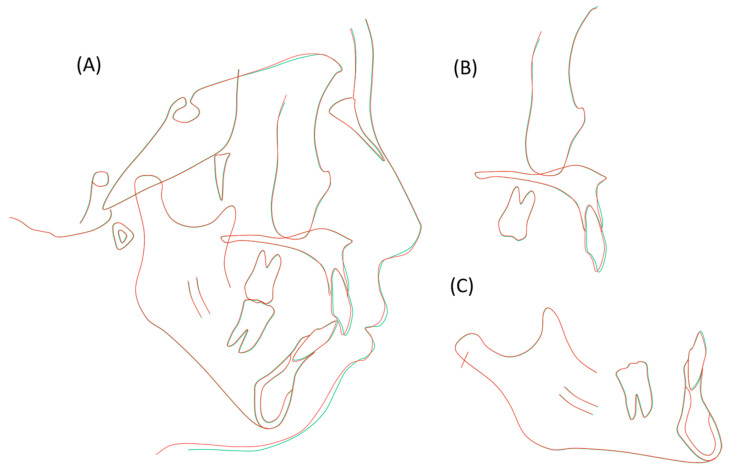
Posttreatment (red; age 28 years, 4 months) and 4-year-and-2-month postretention (green; age 32 years, 6 months) cephalometric tracings superimposed on the internal contour of the anterior wall of the sella turcica (**A**), the anterior contour of the zygomatic process (**B**), and the internal contour of the cortical plate at the inferior border of the symphysis (**C**).

At 4 years and 2 months postretention, her occlusion was relatively stable with a slight relapse of the right canine, and the balanced facial profile was well maintained (Figure 9). The overjet and overbite were well maintained by 2.0 mm (Figure 9). The maxillary and mandibular intermolar widths exhibited no or less changes during retention. The panoramic radiograph and lateral cephalogram revealed little or no change in the denture and skeletal patterns, although the maxillary right first premolar was discolored with a radiolucency at the root apex (Figure 10). The cephalometric analysis revealed that almost no relapse of the maxillary and mandibular incisal inclinations was noted (Figure 10 and Figure 11; Table 1).

## 3. Discussion

In this study, we reported skeletal maxillary expansion treatment with SARME followed by maxillary group distalization with the Anchor-Lock external plate system, which achieved sufficient improvement of a severe maxillary protrusion, and tooth crowding was completed without premolar extraction. Previously, several reports have been published in which skeletal maxillary protrusion with a narrower maxillary arch was treated with orthognathic surgery, such as anterior maxillary alveolar osteotomy and multisegmental LeFort I osteotomy [23,24]. However, these treatment procedures must include premolar extraction, and like the present case, skeletal maxillary protrusion with a narrower maxillary arch and excessive overjet is extremely difficult to treat with only simple maxillary expansion and maxillary group distalization using a skeletal anchorage device alone.

SARME is known to be an effective surgical procedure for maxillary skeletal expansion for the promotion or enhancement of stability in large dentofacial anomalies with >7 mm discrepancy and maxillary transverse deficiency in adults [25]. Anttila et al. [15] found that the feasibility and the long-term stability of SARME were comparable to those of other more invasive osteotomies. Regarding the relapse after SARME, Magnusson et al. [17] reported that maxillary transverse dimensions expanded by SARME decreased significantly within the initial 3 years after SARME and became stable within 6 years after treatment. Gamage and Goss [26] reviewed a case–cohort study of SARME and summarized that intermolar expansion of >6 mm can be achieved with SARME, while more than 60% overexpansion may be required to compensate recurrence. For our patient, we were carried out 8.0 mm of estimated transverse maxillary expansion by SARME, whereas the minimum amount of bilateral maxillary expansion required to correct maxillary protrusion and excessive overjet was 8.0 mm. Moreover, the actual amount of maxillary expansion was 7.0 mm at posttreatment. Taken together, the patient exhibited minimal or no relapse during 5 years after surgery including 16 months of postoperative treatment, indicating long-term stability of skeletal expansion by SARME followed by rigid bone fixation with the Anchor-Lock system.

In general, the distraction device should remain attached to the teeth and palate for approximately 4 to 6 months (retention period) to maintain palatal expansion and allow the bone to heal. After SARME, we also wait about half a year for the bone to heal at the site of the maxillary midpalatal suture. In the present case, we chose to use the Anchor-Lock external plate system [18]. With the use of this system, it is not necessary to wait for a sufficient latency period after SARME [19]. Since we can start distalizing the maxillary molars without the latency period, this system contributes to a reduction in the treatment time.

Although the SARME has many advantages, it also induces many complications: severe hemorrhage, gingival recession, root resorption, nerve injury, infection, pain, and pulp changes in blood flow, sensitivity, and vitality [14,27,28,29]. According to Smeets et al. [28], the incidence of complications after the SARME was more than 50%, and the most common complications were neurosensory disturbances (27.0%) and postoperative pain (13.51%). In particular, the neurosensory disturbances lasting more than 1 year increased with patient’s age significantly. Although a few reports demonstrated that SARME does not affect the health status of the periodontal tissues [30], orthodontists and surgeons should be aware of these complications before performing SARME. In addition, further randomized clinical trials are needed before providing the final conclusions.

The maxillary and mandibular molars’ distalization is often used for the correction and alleviation of mild-to-moderate crowding without causing a detrimental protrusion of the arch. Temporary skeletal anchorage devices, especially orthodontic miniscrews, have been accepted by orthodontists and patients because of their mechanical simplicity and the fact that they do not have a requirement for patient cooperation. Miniscrews allow for a large amount of the maxillary molar distalization without any adverse side effects [31,32]. For instance, it has been reported that an average of 3.78 mm of distal movement is possible with miniscrews [33]. Our patient also achieved 5.0 mm and 6.0 mm distal maxillary molar movements on the right and left sides, respectively. Compared with the distal movement of the maxillary molars, the mandible has greater anatomic constraints on the amount of molar distalization possible. Previously, we proposed the method for predicting the posterior anatomic limit of mandibular molar distalization based on lateral cephalograms and identified the distance (TC-V) between the cervix of the mandibular second molar (TC) and the external oblique line of the mandible (E) as a key measurement for predicting possible mandibular molar distalization [34]. As a result, subjects with TC-V > 0 mm showed approximately 4.0 mm of molar distalization, while subjects with TC-V < 0 mm showed only 1.1 mm of molar distalization. In our patient, the value of TC-V was 1.0 mm, and the total distalization of the right mandibular molars was 4.0 mm. This indicates the importance of calculating the possible mandibular second molar distalization in treatment planning.

## 4. Conclusions

In the present case, a skeletal maxillary protrusion with a narrow maxillary arch, excessive overjet, and severe anterior tooth crowding was successfully treated with SARME and distalization of the maxillary dentitions, avoiding premolar extraction. Rigid bone fixation with the Anchor-Lock system after SARME allows us to reduce treatment time and provides long-term stability of an acceptable occlusion with functional Class I canine and molar relationships. This suggests that SARME followed by the rigid bone fixation may result in a favorable occlusal outcome in the long term without relapse.

## Figures and Tables

**Table 1:** Summary of cephalometric measurements.

	Japanese Adult Female		Pretreatment	Posttreatment	Postretention
Variables	Mean	SD	25 y 0 m	28 y 1 m	32 y 6 m
Skeletal pattern					
SNA	80.8	3.6	82.5	82.0	82.0
SNB	77.9	4.5	74.0	75.5	75.5
ANB	2.8	2.4	**8.5**	**6.5**	**6.5**
Facial angle	84.2	4.4	**76.0**	**77.5**	**77.0**
*Y*-axis	66.1	3.6	**76.0**	**74.0**	**74.0**
Mand. pl./FH	30.5	3.6	**44.5**	**42.5**	**42.5**
Mand. pl./SN	37.1	4.6	**47.0**	**45.0**	**45.0**
Gonial angle	122.1	5.3	**135.0**	**134.5**	**134.5**
Denture pattern					
Occ. pl. to SN	16.9	4.4	18.5	**22.0**	**23.0**
U1 to SN	105.9	8.8	**121.0**	**91.5**	**91.5**
L1 to Mand. pl.	93.4	6.8	**86.5**	95.5	95.0
FMIA	56.0	8.1	49.0	**42.0**	**42.5**
Interincisal angle	123.6	10.6	**108.5**	122.0	122.0

ANB, Anteroposterior positional relation between the maxilla and mandible; SNA, Maxillary position in relation to the anterior cranial base; SNB, Mandibular position in relation to the anterior cranial base; Facial angle, Angle between nasion-pogonion plane and Frankfort horizontal plane; Mand. pl./FH, Angle between mandibular plane and Frankfort horizontal plane; Mand. pl./SN, Angle between mandibular plane and sella-nasion plane; Gonial angle, Angle between mandibular and ramus planes; Occ. Pl. to SN, Angle between occlusal plane and sella-nasion plane; U1 to SN, Axis of maxillary central incisor to sella-nasion plane; L1-Mand. pl., Axis of mandibular central incisor to mandibular plane; FMIA, Angle between mandibular incisal axis and Frankfort horizontal plane; Interincisal angle, angle between maxillary and mandibular central incisal axes. Bold indicates the values deviating from normal ranges.

## Data Availability

Not applicable.
